# Genomic Evidence of *mcr-1.26* IncX4 Plasmid Transmission between Poultry and Humans

**DOI:** 10.1128/spectrum.01015-23

**Published:** 2023-06-26

**Authors:** Ulrike Binsker, Kathrin Oelgeschläger, Bernd Neumann, Guido Werner, Annemarie Käsbohrer, Jens A. Hammerl

**Affiliations:** a Department Biological Safety, German Federal Institute for Risk Assessment, Berlin, Germany; b Institute for Hospital Hygiene, Medical Microbiology and Clinical Infectiology, Paracelsus Medical University, Nuremberg General Hospital, Germany; c Department of Infectious Diseases, Robert Koch Institute, Wernigerode, Germany; d Department for Farm Animals and Veterinary Public Health, Institute of Veterinary Public Health, University of Veterinary Medicine Vienna, Vienna, Austria; Universitat Greifswald

**Keywords:** *Escherichia coli*, colistin, horizontal gene transfer, plasmid-mediated resistance, transfer, *mcr*, IncX4

## Abstract

Colistin is still commonly used and misused in animal husbandry driving the evolution and dissemination of transmissible plasmid-mediated colistin resistance (*mcr*). *mcr-1.26* is a rare variant and, so far, has only been detected in Escherichia coli obtained from a hospitalized patient in Germany in 2018. Recently, it was also notified in fecal samples from a pigeon in Lebanon. We report on the presence of 16 colistin-resistant, *mcr-1.26*-carrying extended-spectrum beta-lactamase (ESBL)-producing and commensal E. coli isolated from poultry samples in Germany, of which retail meat was the most common source. Short- and long-read genome sequencing and bioinformatic analyses revealed the location of *mcr-1.26* exclusively on IncX4 plasmids. *mcr-1.26* was identified on two different IncX4 plasmid types of 33 and 38 kb and was associated with an IS*6*-like element. Based on the genetic diversity of E. coli isolates, transmission of the *mcr-1.26* resistance determinant is mediated by horizontal transfer of IncX4 plasmids, as confirmed by conjugation experiments. Notably, the 33-kb plasmid is highly similar to the plasmid reported for the human sample. Furthermore, we identified the acquisition of an additional beta-lactam resistance linked to a Tn*2* transposon on the *mcr-1.26* IncX4 plasmids of three isolates, indicating progressive plasmid evolution. Overall, all described *mcr-1.26*-carrying plasmids contain a highly conserved core genome necessary for colistin resistance development, transmission, replication, and maintenance. Variations in the plasmid sequences are mainly caused by the acquisition of insertion sequences and alteration in intergenic sequences or genes of unknown function.

**IMPORTANCE** Evolutionary events causing the emergence of new resistances/variants are usually rare and challenging to predict. Conversely, common transmission events of widespread resistance determinants are quantifiable and predictable. One such example is the transmissible plasmid-mediated colistin resistance. The main determinant, *mcr-1*, has been notified in 2016 but has successfully established itself in multiple plasmid backbones in diverse bacterial species across all One Health sectors. So far, 34 variants of *mcr-1* are described, of which some can be used for epidemiological tracing-back analysis to identify the origin and transmission dynamics of these genes. Here, we report the presence of the rare *mcr-1.26* gene in E. coli isolated from poultry since 2014. Based on the temporal occurrence and high similarity of the plasmids between poultry and human isolates, our study provides first indications for poultry husbandry as the primary source of *mcr-1.26* and its transmission between different niches.

## INTRODUCTION

Colistin is used as a last-resort antimicrobial for the treatment of infections with multidrug-resistant Gram-negative bacteria in humans, but it is also widely administered as a therapeutic agent in veterinary medicine for the treatment of gastrointestinal Escherichia coli infection in pigs and poultry (1, 2). The One Health concept recognizes the interconnection between the human, animal, and environmental sectors and the associated risk of spillover of antibiotic-resistant bacteria and/or resistance genes, meaning that, for example, frequent antibiotic use in the animal sector will inevitably affect resistance prevalence in the human and environmental sectors. In light of the One Health approach, which is pursued by the German Antibiotic Resistance Strategy (DART 2020), effort has been made to limit colistin use in veterinary medicine (3). In Germany, sales of colistin in the veterinary sector declined by 53% between 2011 and 2020 as envisaged by the German Antibiotics Minimization Concept (4, 5). However, Germany still ranks 4th among countries in the European Union that sell the most colistin for food-producing animals (6). Calculations of antibiotic use in livestock production in Germany showed that colistin is frequently heavily overdosed especially in poultry husbandry, which may drive the emergence and transmission of colistin-resistant bacteria (7, 8). The ever-increasing demand and large-scale production of retail meat are considered as drivers of resistant bacteria entering the food chain (9). Consequently, contaminated animal-derived food products may serve as a source for consumer exposure to colistin-resistant bacteria.

Colistin resistance in *Enterobacterales* is mainly mediated by the mobile colistin resistance (*mcr*) gene located on transmissible plasmids (10, 11). Since the first report in 2016, 10 different *mcr* genes have been found, of which *mcr-1* is the most common. In addition, 34 variants of *mcr-1* have been described to date with varying prevalence rates. *mcr-1* is often associated with mobile genetic elements (MGE) facilitating transmission across species and habitat boundaries. Particularly, the *mcr-1.1* variant has successfully established itself in diverse genetic backgrounds (plasmid backbones) and bacterial species allowing for dissemination across all sectors around the globe (12, 13). *mcr-1.1* probably originated from livestock animals, indicating a potential zoonotic origin of *mcr-1.1* in humans (11, 14). The impact of livestock in the emergence and transmission of resistance determinants to humans is still understudied and remains controversial (15, 16). Furthermore, the extent to which livestock animals and humans share the same resistance determinants has not been adequately addressed. Whereas most studies focus on the predominant *mcr-1.1* in the animal, human, and environmental sector, studies on the dissemination of rare *mcr-1* variants are lacking. However, rare variants, such as *mcr-1.26* allow for epidemiological tracing-back analysis to identify the origin and transmission dynamics of these genes even beyond species and habitat boundaries. The *mcr*-*1.26* gene was described in 2018 and 2022 in an E. coli isolate from a hospitalized patient in Hesse, Germany, and in fecal samples from a pigeon in Lebanon, respectively (17). Notably, *mcr-1.26* was shown to be linked to IncX4 plasmids, which were shown to be conjugative for the human isolate.

Herein, we report the presence of *mcr*-*1.26* in extended-spectrum beta-lactamase (ESBL)-producing E. coli isolated from healthy poultry and meat thereof in Germany. All poultry isolates described in this study were collected between 2014 and 2022 in the framework of the federal monitoring programs of antimicrobial resistance (AMR) in E. coli. Comparative bioinformatics analyses suggest horizontal transfer of a highly conserved transmissible *mcr-1.26* IncX4 plasmid type within poultry husbandry and demonstrate a high similarity to the plasmid obtained from E. coli from human clinical specimen (17). We further identified a novel *mcr*-*1.26*-IncX4 type that acquired an additional resistance gene (*bla*_TEM_), suggesting that stable *mcr-1.26*-IncX4 plasmids are also subject to evolution, eventually leading to multidrugresistance plasmids. Additionally, conjugative *mcr-1.26*-IncX4 plasmids might be involved in zoonotic transmission via the food chain. Our study provides further evidence for poultry husbandry as the source for the emergence of new *mcr* gene variants and their persistence, indicating a link between the two different niches (animal and human) through the presence of highly conserved *mcr-1.26*-IncX4 plasmids.

## RESULTS

### Poultry food chain as a common source for *mcr-1.26*-carrying E. coli.

The dynamics of antimicrobial resistance are evaluated annually as part of zoonoses surveillance. With the discovery of mobile colistin resistance determinants since 2015, plasmids involved in colistin dissemination are attracting further attention. Selected *mcr-1*-carrying E. coli isolates collected within the annual national monitoring program for zoonoses were analyzed for investigation of the diversity of *mcr*-carrying plasmids. Thereby, 16 isolates were identified to harbor the *mcr-1.26* variant. Notably, all isolates were obtained between 2014 and 2022 from poultry, more specifically from chicken and turkey retail meat as well as chicken and turkey cecal and fecal samples (Table 1). The isolates originate from 10 different German federal states, and metadata do not indicate an epidemiologic association between isolates. Retail meat was the most common source with 9/16 samples from which *mcr-1.26*-carrying E. coli were recovered.

**Table 1:** Overview of relevant genomic and phenotypic characteristics of *mcr-1.26*-harboring E. coli isolates from Germany

Parameter	Isolate																
	14-AB01188	14-AB02757	16-AB00667	16-AB01274	16-AB01348	16-AB01455	16-AB02309	16-AB02976	18-AB01434	18-AB02652	20-AB00574	20-AB00891	20-AB01546	22-AB00280	22-AB00571	22-AB01721	803-18
Source	Turkey, meat^*a*^	Turkey, meat^*a*^	Turkey, cecum^*b*^	Turkey, cecum^*b*^	Turkey, feces^*c*^	Turkey, meat^*a*^	Chicken, meat^*a*^	Broiler, feces^*c*^	Chicken, meat^*a*^	Turkey, cecum^*b*^	Turkey, cecum^*b*^	Chicken, meat^*a*^	Turkey, cecum^*b*^	Chicken, meat^*a*^	Turkey, meat^*a*^	Turkey, meat^*a*^	Human, blood culture
Year	2014	2014	2016	2016	2016	2016	2016	2016	2018	2018	2020	2020	2020	2022	2022	2022	2018
Origin in Germany	Bavaria	Lower Saxony	Saxony	Lower Saxony	Lower Saxony	Mecklenburg-Western Pomerania	Brandenburg	Lower Saxony	Rhineland-Palatinate	Lower Saxony	Lower Saxony	Brandenburg	Lower Saxony	North Rhine-Westphalia	Schleswig-Holstein	Saxony	Hesse
Database accession no.																	
GenBank assembly no.	GCA_029946205.1	GCF_029946215.1	GCA_029946285.1	GCA_029946225.1	GCA_029946265.1	GCA_029946315.1	GCA_029946305.1	GCA_029946345.1	GCA_029946355.1	GCA_029946385.1	GCA_024998065.1	GCA_024998045.1	GCA_029946395.1	GCA_029946425.1	GCA_029946445.1	GCA_030144835.1	GCA_010806905.1
BioProject no.	PRJNA726012	PRJNA726012	PRJNA726012	PRJNA726012	PRJNA726012	PRJNA726012	PRJNA726012	PRJNA726012	PRJNA726012	PRJNA726012	PRJNA726012	PRJNA726012	PRJNA726012	PRJNA726012	PRJNA726012	PRJNA726012	PRJNA605141
BioSample no.	SAMN31082644	SAMN31083434	SAMN31083481	SAMN31083482	SAMN31083483	SAMN31083485	SAMN31083571	SAMN31083737	SAMN31083756	SAMN31083973	SAMN18917895	SAMN18917900	SAMN31083987	SAMN31083989	SAMN31083990	SAMN31953606	SAMN14048832
Phylogroup^*d*^	A	A	B1	A	A	A	B1	B1	A	A	B1	A	A	E	A	A	B1
Serotype^*e*^	O101:H10	O101:H9	O89:H38	O101:H9	O101:H10	O118/O151:H29	O8:H30	O76:H28	O177:H25	O101:H9	O21:H9	O101:H10	O101:H9	O166:H45	O101/O8:H17	O101:H9	O-:H45
(% Identity)	(100:99.84)	(100:98.86)	(100:99.78)	(100:98.86)	(100:99.84)	(100:97.97)	(100:99.94)	(100:100)	(99.69:100)	(100:98.86)	(100:100)	(100:99.92)	(100:98.86)	(100:99.82)	(100:99.9)	(100:98.86)	(-:98.36)
MLST-type^*e*^	744	744	154	744	10	48	1431	224	10	4981	533	10	10	3776	4981	4981	155
cgMLST^*e*^ (% called alleles)	29209 (94.95)	161706 (95.26)	118472 (94.95)	161706 (95.38)	33968 (95.15)	192428 (93.95)	59506 (95.58)	187298 (95.34)	158531 (88.18)	38972 (95.3)	47988 (98.61)	102525 (97.69)	5230 (95.15)	114637 (95.15)	142590 (95.3)	38972 (94.87)	138528 (95.5)
Genome size^*f*^ (bp)	4,939,690	5,101,779	4,892,455	5,050,326	5,031,266	5,198,973	4,956,908	5,155,577	5,069,781	5,008,694	5,389,466	4,768,943	4,979,217	4,946,538	5,045,539	5,233,313	4,921,290
Total no. of genes^*g*^	4,937	5,135	4,915	5,071	5,064	5,343	4,923	5,130	5,203	4,979	5,487	4,662	5,059	4,777	4,996	5,257	4,854
Genetic resistance determinants^*e*^ (% identity)																	
Aminoglycoside	*aph(3′)-Ia* (100)	*aph(3′)-Ia* (100)		*aph(3′)-Ia* (100)		*aadA1* (100)		*aadA1* (100)	*aadA1* (100)	*aadA1* (100)	*aadA1* (100)	*aac(6′)-Ib-cr* (100)		*aac(3)-IId* (99.88)	*aadA1* (100)	*aadA1* (100)	*aadA17* (98.86)
	*aph(3″)-Ib* (100)	*aph(3″)-Ib* (100)		*aph(3″)-Ib* (100)		*aadA2b* (99.87)	*aph(3″)-Ib* (100)	*aph(3″)-Ib* (100)		*aadA2b* (99.87)	*aph(3″)-Ib* (100)		*aph(3″)-Ib* (99.87)	*aadA17* (100)	*aadA2b* (99.87)	*aadA2b* (99.87)	*aph(3″)-Ib* (100)
	*aadA5* (100)	*aadA5* (100)		*aadA5* (100)			*aph(6)-Id* (100)	*aph(6)-Id* (100)			*aph(6)-Id* (100)		*aph(6)-Id* (100)		*aadA5* (100)		*aph(6)-Id* (100)
	*aph(6)-Id* (100)	*aph(6)-Id* (100)		*aph(6)-Id* (100)											*aph(3′)-Ia* (100)		
															*aph(3″)-Ib* (100)		
															*aph(6)-Id* (100)		
Beta-lactam	*bla*_TEM-1B_ (100)	*bla*_TEM-135_ (99.88)	*bla*_TEM-1B_ (100)	*bla*_TEM-135_ (99.88)	*bla*_TEM_ (99.87)	*bla*_TEM-1B_ (100)	*bla*_CTX-M-15_ (100)	*bla*_CTX-M-1_ (100)	*bla*_TEM-1B_ (100)	*bla*_CTX-M-15_ (100)	*bla*_CTX-M-27_ (100)	*bla*_CTX-M-15_ (100)	*bla*_TEM-135_ (99.88)	*bla*_TEM-1B_ (100)	*bla*_CTX-M-15_ (100)	*bla*_CTX-M-15_ (100)	*bla*_TEM-1B_ (100)
					90% coverage)		*bla*_TEM-1B_ (100)	*bla*_TEM-1B_ (100)			*bla*_TEM-135_ (99.88)	*bla*_OXA-1_ (100)		*bla*_CTX-M-55_ (100)	*bla*_TEM-135_ (99.84)		
Colistin	*mcr-1.26* (100)	*mcr-1.26* (100)	*mcr-1.26* (100)	*mcr-1.26* (100)	*mcr-1.26* (100)	*mcr-1.26* (100)	*mcr-1.26* (100)	*mcr-1.26* (100)	*mcr-1.26* (100)	*mcr- 1.26* (100)	*mcr-1.26* (100)	*mcr-1.26* (100)	*mcr-1.26* (100)	*mcr-1.26* (100)	*mcr-1.26* (100)	*mcr-1.26* (100)	*mcr-1.26* (100)
Folate pathway antagonist	*dfrA17* (100)	*dfrA17* (100)		*dfrA17* (100)			*dfrA14* (100)	*dfrA1* (99.79)	*dfrA1* (99.79)		*dfrA1* (99.79)			*dfrA14* (100)	*dfrA17* (100)		*dfrA14* (100)
	*sul1* (100)	*sul1* (100)		*sul1* (100)		*sul3* (100)	*sul2* (100)	*sul1* (100)	*sul1* (100)	*sul3* (100)	*sul1* (100)			*sul3* (100)	*sul2* (99.88)	*sul3*	*sul2* (100)
	*sul2* (100)	*sul2* (100)		*sul2* (100)				*sul2* (100)			*sul2* (99.88)				*sul3* (100)		
Lincosamide									*Inu*(F) (100)					*Inu*(F) (100)			*Inu*(F) (100)
Macrolide	*mph*(A) (100)	*mph*(A) (100)		*mph*(A) (100)				*mph*(B) (100)									
	*mdf*(A) (99.92)	*mdf*(A) (99.92)	*mdf*(A) (98.46)	*mdf*(A) (99.92)	*mdf*(A) (99.92)	*mdf*(A) (99.92)	*mdf*(A) (98.95)	*mdf*(A) (98.46)	*mdf*(A) (98.86)		*mdf*(A) (99.03)		*mdf*(A) (99.92)				*mdf*(A) (98.46)
Phenicol	*catA1* (99.85)	*catA1* (99.85)		*catA1* (99.85)		*cmlA1* (99.92)		*catA1* (99.85)		*cmlA1* (99.92)	*florR* (98.19)				*cmlA1* (99.92)	*cmlA1* (99.92)	
Quinolone	*gyrA*_S83L	*gyrA*_S83L		*gyrA*_S83L	*gyrA*_S83L	*gyrA*_S83L	*gyrA*_S83L	*gyrA*_S83L	*gyrA*_S83L	*gyrA*_S83L	*gyrA*_S83L	*gyrA*_S83L			*gyrA*_S83L	*gyrA*_S83L	*gyrA*_S83L
				*gyrA*_D87N	*gyrA*_D87N		*gyrA*_D87N	*gyrA*_D87N		*gyrA*_D87N	*gyrA*_D87N	*gyrA*_D87N			*gyrA*_D87N	*gyrA*_D87N	
				*parC*_A56T	*parC*_S80I		*parC*_S80I	*parC*_S80I		*parC*_S80I	*parC*_S80I	*parC*_S80I			*parC*_S80I	*parC*_S80I	
				*parC*_S80I			*parE*_S458A			*parE*_S458A		*parE*_S458A			*parE*_S458A	*parE*_S458A	
							*qnrS1* (100)							*qnrS1* (100)			
Tetracycline	*tet*(B) (100)	*tet*(B) (100)		*tet*(B) (100)		*tet*(A) (100)	*tet*(A) (100)	*tet*(A) (100)	*tet*(A) (100)		*tet*(A) (99.92)	*tet*(B) (100)	*tet*(A) (99.92)		*tet*(B) (100)		*tet*(A) (100)
											*tet*(B) (100)						
Amino acid exchanges in colistin resistance proteins			PmrB D283G^*h*^ PmrB Y358N^*i*^			PmrB H2R^*h*^	PmrB D283G^*h*^ PmrB Y358N^*i*^	PmrB D283G^*h*^ PmrB Y358N^*i*^ PmrA G144S^*h*^			PmrB D283G^*h*^ PmrB Y358N^*i*^			PmrB H2R^*h*^ PmrB A242T^*j*^ PmrB D283G^*h*^ PmrB V351I^*i*^			PmrB D283G^*h*^ PmrB Y358N^*i*^

a Raw meat from retail.

b Cecum samples were taken during the slaughter process.

c Feces samples were taken from the animal farm.

d Phylogroup was determined using the web-based tool ClermonTyper (http://clermontyping.iame-research.center/).

e *In silico* analysis was performed using web-based tools SeroTypeFinder 2.0.1, MLST-Finder 2.0.9, cgMLSTFinder 1.2, and ResFinder 4.1 of the Center for Genomic Epidemiology (www.genomicepidemiology.org). The percentage of identity of the target sequence compared to the reference is given in parentheses.

f The genome size was determined using the AQUAMIS pipeline (https://gitlab.com/bfr_bioinformatics/AQUAMIS/).

g The total gene count was obtained using the NCBI Prokaryotic Genome Annotation Pipeline (PGAP).

h Polymorphism does not contribute to colistin resistance.

i Reported polymorphism but its association with colistin resistance is unknown and not experimentally confirmed.

j Unknown contribution to colistin resistance.

### Phenotypic antimicrobial resistance profiles.

With the exception of three substances (amikacin, meropenem, tigecycline), the isolates demonstrated resistance phenotypes to a multitude of antibiotics, including highly and critically important antimicrobials. All E. coli isolates were phenotypically resistant to colistin (MIC ≥ 4 mg/L) followed by ampicillin (15/16, 94%), ciprofloxacin/nalidixic acid (13/16, 81%), sulfamethoxazole (12/16, 75%), tetracycline (11/16, 69%), cefotaxime/ceftazidime (9/16, 56%), trimethoprim (9/16, 56%), chloramphenicol (9/16, 56%), and gentamicin (1/16, 6%) (Table 2; see also Fig. S1 in the supplemental material). Notably, the *mcr-1.26*-positive human clinical E. coli isolate 803-18 showed a similar resistance phenotype (17).

**TABLE 2:** Antimicrobial susceptibility profiles of *mcr-1.26* donor strains and transconjugants^*a*^

Parameter	Isolate (MIC in mg/L)														
	AK	AMP	AZI	CHL	CIP	COL	FOT^*b*^	GEN	MERO	NAL	SMX	TAZ^*b*^	TET	TGC	TMP
Range tested	4–128	1–32	2–64	8–64	0.015–8	1–16	0.25–4	0.5–16	0.03–16	4–64	8–512	0.25–8	2–32	0.25–8	0.25–16
MIC sensitive	≥16	≤8	NA	≤8	≤0.25		≤1	≤4	≤1	≤16	≤256	≤ 4	≤ 4	NA	≤8
MIC intermediate	32	16	NA	16	0.5	≤2	2	8	2			8	8	NA	
MIC resistant	≥64	≥32	NA	≥32	≥1	≥4	≥4	≥16	≥4	≥32	≥512	16	≥16	NA	≥16
*mcr-1.26*															
14-AB01188	≤4	>32	64	>64	>8	8	≤0.25	≤0.5	≤0.03	>64	>512	≤0.25	>32	≤0.25	>16
Conjugant 1188	≤4	8	4	≤8	0.03	4	≤0.25	≤0.5	≤0.03	≤4	≤8	0.5	≤2	≤0.25	≤0.25
14-AB02757^*c*^	≤4	>32	16	>64	8	8	≤0.25	1	≤0.03	>64	>512	≤0.25	>32	≤0.25	>16
16-AB00667	≤4	>32	8	≤8	0.03	4	≤0.25	≤0.5	≤0.03	≤4	≤8	≤0.25	≤2	≤0.25	≤0.25
Conjugant 667	≤4	8	8	≤8	0.03	8	≤0.25	1	≤0.03	≤4	≤8	0.5	≤2	≤0.25	≤0.25
16-AB01274	≤4	>32	64	>64	8	8	≤0.25	≤0.5	≤0.03	>64	>512	≤0.25	>32	≤0.25	>16
Conjugant 1274	8	4	8	≤8	0.03	8	≤0.25	≤0.5	≤0.03	≤4	≤8	0.5	4	0.5	≤0.25
16-AB01348	≤4	4	8	≤8	>8	8	≤0.25	≤0.5	≤0.03	>64	≤8	≤0.25	≤2	≤0.25	≤0.25
Conjugant 1348	8	8	4	≤8	0.03	8	≤0.25	2	≤0.03	≤4	≤8	0.5	≤2	≤0.25	≤0.25
16-AB01455	8	>32	4	64	0.5	8	≤0.25	1	≤0.03	>64	>512	≤0.25	>32	≤0.25	≤0.25
Conjugant 1455	≤4	8	4	≤8	0.03	4	≤0.25	≤0.5	≤0.03	≤4	≤8	0.5	4	≤0.25	≤0.25
16-AB02309	8	>32	8	≤8	>8	8	>4	1	≤0.03	>64	>512	8	>32	≤0.25	>16
Conjugant 2309	≤4	8	8	≤8	0.03	4	≤0.25	≤0.5	≤0.03	≤4	≤8	0.5	≤2	0.5	≤0.25
16-AB02976	≤4	>32	8	>64	>8	8	>4	≤0.5	≤0.03	>64	>512	2	>32	≤0.25	>16
Conjugant 2976	≤4	>32	4	≤8	≤0.015	8	>4	≤0.5	≤0.03	≤4	>512	4	>32	≤0.25	≤0.25
18-AB01434	≤4	>32	4	≤8	0.25	8	4	2	≤0.03	>64	>512	1	>32	0.5	>16
Conjugant 1434	≤4	8	8	≤8	≤0.015	4	≤0.25	≤0.5	≤0.03	≤4	≤8	≤0.25	4	≤0.25	≤0.25
18-AB02652	≤4	>62	8	64	>8	8	>4	≤0.5	≤0.03	>64	>512	8	4	≤0.25	≤0.25
Conjugant 2652	≤4	8	4	≤8	0.03	8	≤0.25	≤0.5	≤0.03	≤4	≤8	0.5	4	≤0.25	≤0.25
20-AB00574	≤4	>32	4	>64	8	4	>4	1	≤0.03	>64	>512	2	>32	≤0.25	>16
Conjugant 574	≤4	>32	4	≤8	≤0.015	8	≤0.25	1	≤0.03	≤4	≤8	0.5	4	≤0.25	≤0.25
20-AB00891	≤4	>32	16	≤8	>8	8	>4	1	≤0.03	>64	16	8	>32	0.5	0.5
Conjugant 891	≤4	4	4	≤8	≤0.015	8	≤0.25	1	≤0.03	≤4	≤8	0.5	8	≤0.25	≤0.25
20-AB01546	≤4	>32	8	≤8	≤0.015	8	≤0.25	≤0.5	≤0.03	≤4	≤8	≤0.25	>32	0.5	≤0.25
Conjugant 1546	≤4	>32	4	≤8	0.03	4	≤0.25	≤0.5	≤0.03	8	≤8	0.5	4	≤0.25	≤0.25
22-AB00280	≤4	>32	4	≤8	0.5	8	>4	>16	≤0.03	≤4	>512	8	≤2	≤0.25	>16
Conjugant 280	≤4	8	4	≤8	≤0.015	8	≤0.25	≤0.5	≤0.03	≤4	≤8	0.5	4	≤0.25	≤0.25
22-AB00571	≤4	>32	8	64	>8	8	>4	1	≤0.03	>64	>512	8	>32	≤0.25	>16
Conjugant 571	≤4	>32	4	≤8	0.03	4	≤0.25	≤0.5	≤0.03	≤4	≤8	1	4	≤0.25	≤0.25
22-AB01721	≤4	>32	8	64	>8	4	>4	≤0.5	≤0.03	>64	>512	8	≤2	≤0.25	≤0.25
Conjugant 1721	≤4	8	4	≤8	≤0.015	4	≤0.25	1	≤0.03	≤4	≤8	0.5	8	≤0.25	≤0.25
Negative control															
SAZ^r^ E. coli J53	≤4	8	8	16	≤0.015	≤1	≤0.25	1	≤0.03	≤4	≤8	0.5	≤2	≤0.25	≤0.25

a MIC, minimal inhibitory concentration; AK, amikacin; AMP, ampicillin; AZI, azithromycin, CHL, chloramphenicol; CIP, ciprofloxacin; COL, colistin; FOT, cefotaxime; GEN, gentamicin; MERO, meropenem; NAL, nalidixic acid; SMX, sulfamethoxazole; TAZ, ceftazidime; TET: tetracycline; TGC, tigecycline; NA, not available; TMP, trimethoprim. Microbiological resistance profiles were determined using the broth microdilution method according to CLSI. Concentration ranges and CLSI criteria for antimicrobials are shown.

b For FOT (MIC 0.5 mg/L) and TAZ (MIC 1.0 mg/L), cutoff values described in Commission Implementing Decision 2020/1729/EU, specifically adapted for the EU-wide harmonized evaluation of ESBL resistance in food-derived E. coli, were applied. For quality assurance of conjugation experiments, the sodium azide-resistant (SAZ^r^) E. coli J53 recipient strain was included as a reference in AST measurements. Resistances in donor strains are marked in light gray. Transferred resistances are marked in dark gray.

c No transconjugants could be obtained from isolate 14-AB02757.

### Genetic heterogeneity of isolates indicates horizontal transmission of *mcr-1.26*.

**FIG 1:**
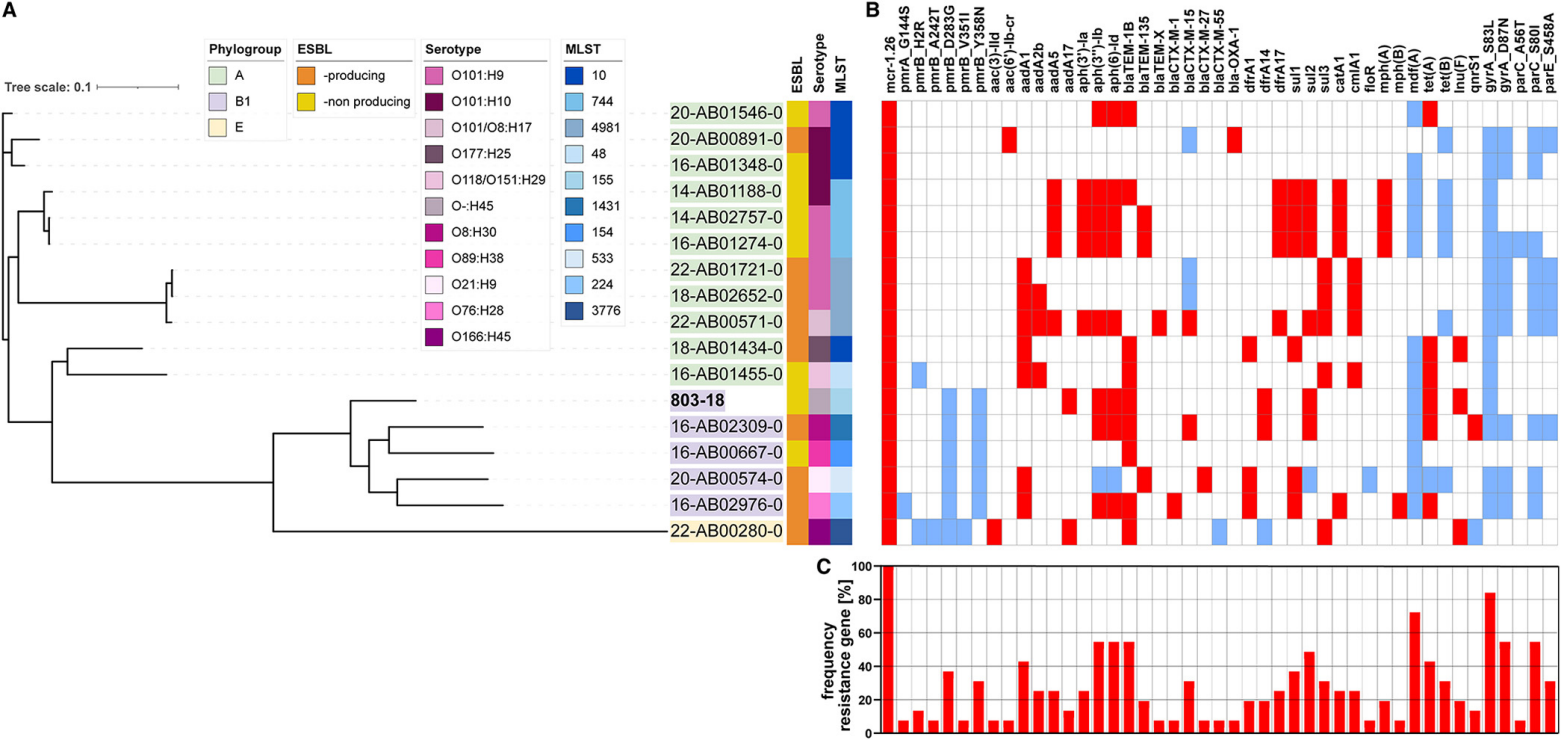
Genetic relatedness and antibiotic resistance profiles of *mcr*-*1.26*-positive E. coli from poultry and human in Germany. (A) A maximum likelihood phylogenetic tree based on 3,722,829 SNPs was constructed with the web-based tool CSIPhylogeny 1.4 of the Center for Genomic Epidemiology (CGE) (www.genomicepidemiology.org) using the human clinical isolate 803-18 as reference. Background of isolate names are colored according to their phylogenetic group, which was determined by ClermonTyper (http://clermontyping.iame-research.center/). Serotype and MLST were identified by SeroTypeFinder 2.0.1 and MLST-Finder 2.0.9 of CGE. (B) Genetic antibiotic resistance profiles of isolates were obtained by using ResFinder 4.1 of CGE. The predicted location of genes in the chromosome (blue) and on plasmids (red) is shown. (C) Percentage frequency of resistance genes. Unless otherwise stated, all programs were used with the default settings.

In order to determine a possible clonal spread of *mcr-1.26*-carrying E. coli or horizontal transmission of the *mcr-1.26* resistance determinant, the phylogenetic relationship of 17 isolates, including the human clinical isolate, was analyzed. Three different E. coli phylogroups were present in the analyzed population, of which phylogroup A was the most common followed by phylogroup B1, which included the human isolate 803-18 (Fig. 1A). The E. coli population studied is characterized by 11 different serotypes and 10 different sequence types (STs), reflecting the overall genomic diversity of the isolates. The majority of sequence types have been described to be present in humans and domestic animals and some belong to pandemic lineages (18–24). Conversely, ST155 of the human clinical isolate 803-18 has been commonly found in poultry samples (19, 25). Notably, two clusters contain two closely related isolates each (14-AB02757 and 16-AB01274; 18-AB02652 and 22-AB01721) represented by an identical core genome multilocus sequence type (cgMLST) (Table 1). Furthermore, these four isolates were obtained from turkey. The single-nucleotide polymorphism (SNP)-based phylogenetic tree using the genome of the human clinical isolate 803-18 as reference showed a substantial diversity of all 17 isolates and covered 75.6% of the reference (Fig. 1A; see also Fig. S2 in the supplemental material).

The virulence profiles of all strains are overall comparable, but three strains possessed additional genes encoding adhesive fimbriae (P fimbriae, *afa*) or a *chu* locus, which are common in pathogenic E. coli (see Table S2 in the supplemental material) (26, 27).

Based on the genetic heterogeneity of the isolates, colistin resistance is likely associated with a mobile genetic element that allows horizontal transmission of the *mcr-1.26* gene. In order to investigate the transferability of the colistin resistance, conjugation assays were performed. Intraspecies transmission of the colistin resistance could be confirmed by molecular detection of *mcr-1*-like genes in transconjugants. The obtained transconjugants showed equal MIC values toward colistin as the corresponding wild-type strains (Table 2). The *mcr-1*-like genes were transferred with a frequency of 1.5 × 10^3^ to 2.1 × 10^5^. The results suggest horizontal gene transfer of *mcr-1.26* rather than clonal spread of colistin-resistant isolates.

### Characterization of the resistome.

Besides *mcr-1.26*, all strains harbored several additional resistance genes and chromosomal mutations leading to antimicrobial resistance (AMR) and a multidrug resistance phenotype. Resistome analysis revealed 45 different resistance determinants in the E. coli population studied, of which isolates 20-AB00574 (obtained from cecum) and 22-AB00571 (obtained from meat) harbored the most resistance genes (*n* = 18) (Fig. 1B). Notably, missense mutations in PmrA and PmrB, a two-component system involved in lipid A modification and thus colistin resistance, were found in six different strains (Table 1). However, mutations have not been experimentally confirmed to cause colistin resistance and have been reported also in sensitive strains (1, 28). Eight different genes conferring resistance to β-lactams were detected in nine isolates, whose ESBL phenotype was confirmed by antimicrobial susceptibility testing (AST). Following *mcr-1.26*, the chromosomal mutations *gyrA*_S83L (82.35%) and *mdf*(A) (70.59%) were the most abundant resistance-associated determinants (Fig. 1C).

### *mcr-1*-like genes are encoded on different conjugative IncX4 plasmid types.

**FIG 2:**
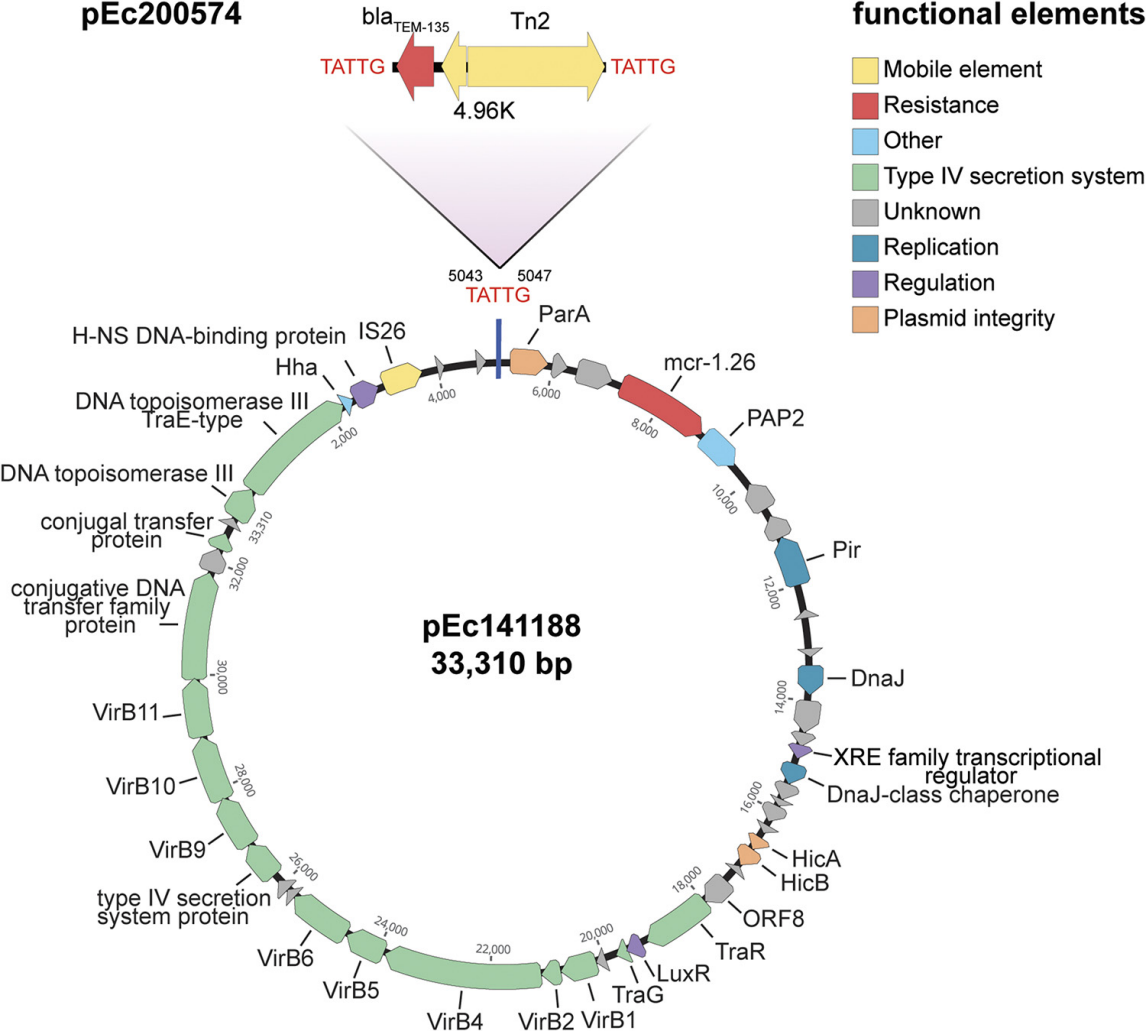
Schematic representation of two *mcr-1.26*-harboring IncX4 plasmid prototypes. pEc141188 (below) represents the basic structure of *mcr-1.26* carrying IncX4 plasmids present in 14 E. coli isolates from poultry and human. Plasmid pEc200574 (above) differs from pEc141188 by insertion of a 4,960-bp Tn*2* element containing a *bla*_TEM-135_ gene and was found in three E. coli isolates. The transposon was inserted at an intergenic TATTG sequence, resulting in the formation of 5-bp direct repeats flanking the transposon.

*In silico* analysis revealed the presence of at least three different incompatibility (Inc) groups in each strain (see Table S2). The *mcr-1.26* genes were located on IncX4 plasmids in all isolates and could be associated with an IS6-like element encoding an IS26 family transposase as previously observed for the human isolate 803-18 (17). We noticed that four transconjugants acquired additional resistance to ampicillin (Table 2). *In silico* analyses and molecular investigation revealed the colocalization of *mcr-1.26* with a *bla*_TEM_ gene in three isolates (see Fig. S3 in the supplemental material). This indicated at least two different IncX4 plasmid types carrying *mcr-1*-like genes, which was confirmed by hybrid assembly of IncX4 plasmids from isolate 14-AB01188 (pEc141188) and 20-AB00574 (pEc200574) (Table 3). A 33,310-bp plasmid was reconstructed for isolate 14-AB01188, carrying functions for their spread and maintenance (i.e., a *hicAB* toxin-antitoxin system) (Fig. 2). BLAST analyses revealed high similarity (99.96%; coverage, 99%; harbors *mcr-1.1*) with a plasmid of similar size (GenBank accession number CP092316.1) obtained from Salmonella enterica (Table 3). In contrast, hybrid assembly generated a 38,265-bp IncX4 plasmid for isolate 20-AB00574. A *bla*_TEM-135_ gene exhibiting an identity of 99.84% to 99.88% was found to have been integrated upstream of *mcr-1.26* by the insertion of a Tn*2* element (Fig. 2). This element integrated at a TATTG sequence in an intergenic region, which resulted in the formation of 5-bp direct repeats flanking the transposon. Sequence comparisons of pEc200574 did not yield a clear result in the NCBI database but predicted similarity to two different plasmids (Table 3). The first match had an identity of 99.99% but covered only 87% of the template sequence (GenBank accession number LR882927.1; carries *mcr-1.26*). The second match showed a much larger plasmid of 95,202 bp, with a coverage of 100% and identity of 99.98% when compared to the reference sequence (GenBank accession number MT929289.1; carries *mcr-1.1*).

**TABLE 3:** Comparison of *mcr-1.26*-harboring IncX4 prototypes^*a*^

Parameter	Plasmid name		
	pEc141188	pEc200574	
Size (bp)	33,310	38,265	
No. of strains detected	14	3 (20-AB00574; 20-AB01546; 22-AB00571)	
Mobile genetic element finder			
Resistance genes (GenBank accession no.)	*mcr-1.26* (NG_068217)	*mcr-1.26* (NG_068217) and *bla*_TEM-135_ (GQ896333)	
Mobile genetic elements (GenBank accession no.)	IS*26* (X00011)	IS*26* (X00011) and Tn*2* (HM749967.1)	
Nucleotide blast			
Max score	61,420	31,964	31,953
Total score	63,924	64,034	78,532
Query coverage (%)	99	87	100
E value	0.0	0.0	0.0
Identity (%)	99.96	99.99	99.98
Acc length (bp)	33,301	33,310	95,202
GenBank accession	CP092316.1	LR882927.1	MT929289.1
Host	Salmonella enterica	E. coli	E. coli

a *In silico* analysis was performed using pEc141188 and pEc200574 as reference in the web-based tools MGEFinder 1.0.2 of the Center for Genomic Epidemiology (www.genomicepidemiology.org; accessed 30 November 2022) and BLASTN from NCBI (https://blast.ncbi.nlm.nih.gov/Blast.cgi; accessed 16 November 2022).

Sequence alignments of trimmed raw reads of poultry and human isolates using pEc141188 and pEc200574 as reference confirmed two different types of *mcr*-carrying IncX4 plasmids within the E. coli population. Plasmid pEc141188 was found in 14 isolates, including the human isolate, whereas pEc200574 was found in three different E. coli isolates obtained from turkey (Table 3). Subsequent analysis of SNPs revealed highly conserved regions in both plasmid types (see Fig. S4 in the supplemental material). All other coding sequences contained synonymous and nonsynonymous SNPs with varying predicted effect on protein function. However, it seems that the SNPs had no obvious impact on the functionality of the plasmid and its transmissibility.

Based on the finding that LR882927.1 carried *mcr-1.26*, we were interested in whether additional *mcr-1.26* genes have been reported in public databases (GenBank accessed 13 January 2023). We identified a total of three reports of *mcr-1.26* genes, which were located on IncX4 plasmids originating from E. coli and also highly similar to pEc141188 (see Table S3 in the supplemental material). Interestingly, E. coli was isolated from a human in the Netherlands (GenBank accession number LR882927.1) and from raw retail turkey meat in Czech Republic (GenBank accession numbers MT929276.1 and MT929278.1), both countries neighboring Germany (29).

## DISCUSSION

The high prevalence of *mcr*-carrying *Enterobacterales* in livestock production and the spillover of resistant bacteria and *mcr* genes into the human and environmental sectors contribute to the global antimicrobial resistance crisis. The estimated global prevalence of *mcr* genes in *Enterobacterales* is 4.7% (47 countries across 6 continents) (13). Animal husbandry is the most significant contributor, with reported prevalences ranging from 1.8% to 63% in poultry and 0.35% to 98.0% in pigs, followed by the human sector with prevalence rates of 0.05% to 4.73% and the environmental sector with prevalence rates ranging from 0.8% to 2.4% (12). In Germany, prevalence rates of *mcr*-positive *Enterobacterales* varied from 4.7% to 8.7% for poultry, from 0.4% to 9.9% for pigs, and 1.8% for wild boar. A recent study covering different stages of the meat production chain in Germany determined an overall prevalence of *mcr-1*-positive *Enterobacterales* of 18%, with the highest prevalence of 43% at a poultry slaughterhouse (30).

The first *mcr-1.26*-positive E. coli isolate was isolated from turkey retail meat in 2014, which coincides with the introduction of mandatory susceptibility testing to colistin for bacteria isolated from food-producing animals implemented in the European Union (31). Therefore, it is possible that *mcr-1.26*-positive E. coli were present in poultry before 2014. Thereafter, *mcr-1.26* E. coli were found at biennial intervals, as the monitoring plan specified poultry sampling every 2 years. Notably, *mcr-1.26* has also been found in samples outside of Germany, an E. coli isolated from a human in Netherlands and two E. coli obtained from retail meat in Czech Republic, which had been imported from Germany. To date, *mcr-1.26* has not been reported in bacterial species other than E. coli or in other animal species. It is worth mentioning that during the BLASTN search, we noticed that other hits had an incomplete start codon, lacking the first two nucleotides, which is crucial to distinguish between *mcr-1.26* and *mcr-1.1* and eventually leading to an underestimation of *mcr-1.26* prevalence (17).

The isolates carrying *mcr-1.26* in this study comprised a variety of different phylogroups, STs, and serotypes, indicating horizontal gene transfer of *mcr*-carrying plasmids rather than clonal spread, although these findings are based on a limited number of 16 isolates. Up to present, 15 different Inc-type plasmids associated with *mcr-1* have been documented (12). Most plasmids are transferable, of which IncX4, IncHI2, and IncI2 are predominant worldwide (32). In the current study, *mcr-1.26* was located exclusively on conjugative IncX4 plasmids. This result is consistent with findings of IncX4 plasmids carrying *mcr-1.26* in the human isolates from Germany and Netherlands as well as isolates from turkey meat from Czech Republic. However, we identified two different types of IncX4 plasmids in our E. coli population. *mcr-1*-harboring IncX4 plasmids with no additional resistance genes seem to disseminate increasingly among *Enterobacterales*, such as E. coli, Salmonella enterica, and Klebsiella pneumoniae, of animal, human, and environmental origin. Comparative analysis to pEc141188 revealed a ubiquitous presence of highly similar plasmids, indicating its genetic stability as well as its successful global dissemination into different sectors.

The presence of an additional *bla*_TEM-135_ gene in pEc200574 suggests that IncX4 plasmids, despite their stability, are also subject to evolution. No similar plasmids could be found in the NCBI database. Interestingly, an IncX4-IncI2 hybrid plasmid of 95,202 bp (MT929289.1) encoded *mcr-1.1* and the *bla*_TEM-135_-Tn*2* cassette, which was isolated from raw retail meat in Czech Republic originally imported from Germany (29). The fact that pEc200574 was present in E. coli of different phylogroups and ST groups highlights the transmissible nature of this IncX4 plasmid type, thus contributing to the spread of multidrug resistance. It remains subject of future investigations whether this plasmid type will disseminate further in turkey husbandry or even into other ecological niches. Both types of IncX4 plasmids contained a *hicAB* toxin-antitoxin system, which contributes to plasmid maintenance (33). The presence of *hicAB* on *mcr-1*-IncX4 plasmids has been described previously and may be an explanation for the successful dissemination of the plasmid type (34, 35).

With our study, we have confirmed the transmissible nature of IncX4 plasmids and their role in the spread of *mcr-1* genes. Interestingly, *mcr-1.26*-IncX4 plasmids have disseminated in two different poultry species, chicken and turkey. Furthermore, we show that retail meat is a common reservoir for *mcr-1.26*-positive E. coli in Germany. This observation is in agreement with previous studies reporting the presence of the *mcr-1*-carrying IncX4 plasmids in *Enterobacterales* from retail meat in different countries, pointing out the significance of food-producing animals and retail meat as reservoirs of *mcr-1*-carrying bacteria and a potential exposure risk to consumers (29, 36–44).

Spontaneous alteration of the coding sequence that do not affect the protein function or the extensive use of colistin in poultry production in Germany may have been a driver for the evolution of the *mcr-1* gene and eventually resulted in the emergence of *mcr-1.26* (7, 8). Colistin is generally administered to the entire flock for disease treatment or metaphylaxis purposes, which may have ensured the persistence of *mcr-1.26* in poultry husbandry in Germany, thus providing a source for *mcr-1.26* transmission into the food chain. Additionally, Germany is one of the largest producers and exporters of poultry and meat thereof in the European Union, which may have led to transnational transmission of *mcr-1.26* and the detection of *mcr-1.26* E. coli on retail meat in Czech Republic (45). Thus, international trade plays a further role in the spread of resistant bacteria.

The study of the transmission routes of colistin-resistant bacteria is complicated by the fact that resistance genes are often transmitted by mobile genetic elements. There is a possibility of transmission of promiscuous IncX4 plasmids containing *mcr-1.26* to other bacterial hosts in diverse environmental conditions. Furthermore, *mcr-1.26*-IncX4 plasmids have been found across distantly related bacterial strains from human and animal origin, which, however, does not exclude a possible zoonotic transmission chain. In line with other research, our results indicate a spillover of *mcr-1*-like genes into different One Health sectors, highlighting the need to reduce colistin pressure through a One Health approach, both by reducing the use of colistin in poultry husbandry and by applying structural and medical cost-effective alternatives to maintain poultry production.

## MATERIALS AND METHODS

### Background of isolates.

E. coli isolates were collected in the framework of the annual national monitoring program for Germany established by the German Federal Institute for Risk Assessment (BfR), the Federal Office for Consumer Protection and Food Safety (BVL), and the authorities of the federal states to comply with Directive 2003/99/EC and commission implementing decisions (CID) 2013/652/EU and 2020/1729/EU. Selected isolates carrying *mcr-1*, as confirmed by PCR, were further characterized by whole-genome sequencing (WGS), of which 16 isolates harbored *mcr-1.26*. The study conducted was not a prevalence analysis. E. coli from this study were isolated from chicken and turkey meat as well as broiler and turkey cecal and fecal samples by regional laboratories and subjected to species confirmation (i.e., growth on indicator media, matrix-assisted laser desorption ionization–time of flight [MALDI-ToF]) and antimicrobial susceptibility testing (AST) at the National Reference Laboratory for Antimicrobial Resistance (NRL-AR) hosted at the BfR, as previously described (46).

### Antimicrobial susceptibility testing.

Susceptibility testing of E. coli isolates to antimicrobial agents was conducted by broth microdilution according to the guidelines of the Clinical and Laboratory Standards Institute (CLSI) M07 (11th ed. M07-A10) (47, 48). A commercial Sensititre system (EUVSEC3; Thermo Scientific, Meerbusch, Germany) with a harmonized European panel of antimicrobials containing 15 antimicrobial substances of 12 antimicrobial classes (amikacin, ampicillin, azithromycin, cefotaxime, ceftazidime, chloramphenicol, ciprofloxacin, colistin, gentamicin, meropenem, nalidixic acid, sulfamethoxazole, tetracycline, tigecycline, and trimethoprim) was used in concentration ranges described in commission implementing decision 2020/1729/EU (49). For quality assessment, the E. coli isolate ATCC 25922 and sodium azide-resistant (SAZ^r^) E. coli J53 was included as a reference in AST measurements.

### Whole-genome, Sanger sequencing, and bioinformatics analyses.

For WGS, bacterial genomic DNA from liquid overnight cultures was isolated using the PureLink genomic DNA preparation minikit (Invitrogen GmbH, Darmstadt, Germany) according to the manufacturer’s instructions. The Nextera DNA Flex library prep kit was used for DNA library preparation as previously published followed by a paired-end (2 × 150) sequence determination on a NextSeq 500 device (Illumina, Inc., San Diego, CA, USA) (50). Subsequently, raw reads were trimmed and assembled using the Aquamis pipeline (https://gitlab.com/bfr_bioinformatics/AQUAMIS/; accessed November 2022 and January 2021) (51) by applying quality control (QC) thresholds as follows: number of contigs (≥0 bp), ≤605; number of contigs (≥1,000 bp), ≤203; assembly coverage depth, >50; and *N*_50_ value in base pairs (indicator of average contig size and assembly quality), >72,925 (see Table S1 in the supplemental material). For plasmid analyses, isolates 14-AB01188 and 20-AB00574 were additionally subjected to long-read sequencing and used as templates for reference mapping-based approaches. Therefore, Oxford Nanopore Technology (ONT) sequencing libraries were sequenced on a MinIon Mk1C device followed by a hybrid assembly using Unicycler v0.4.8 as previously described (52).

In order to confirm the *mcr-1.26* variant, commercial Sanger sequencing of *mcr-1* gene amplicons was performed for all isolates (Eurofins Genomics, Ebersberg, Germany) using the primers and PCR protocol as described (17).

Annotation of the bacterial genomes was performed using the automated Prokaryotic Genome Annotation Pipeline (PGAP) (National Center for Biotechnology Information) (53). Phylogenetic analysis of *mcr*-carrying E. coli was conducted using a reference genome-based, single-nucleotide polymorphism (SNP) strategy with CSIPhylogeny 1.4 under default settings (54). The phylogenetic tree was visualized using the iTOL 6.6 software (55). Isolates were characterized *in silico* using the web-based tools provided by the Center for Genomic Epidemiology under the default settings (www.genomicepidemiology.org) (Table 1). The phylogroup was determined *in silico* using the web-interface ClermonTyper (http://clermontyping.iame-research.center/) (56).

Identification of different IncX4 plasmid types and analyses of SNPs was performed with Geneious Prime 2020.2.2 software under default settings using the hybrid reference assemblies for plasmid genomes pEc141188 (isolate 14-AB01188) and pEc200574 (isolate 20-AB00574). A minimum variant frequency threshold of 50% was used for SNP identification. Plasmid-encoded genes were annotated using PATRIC (The Bacterial and Viral Bioinformatics Resource Center; https://www.bv-brc.org/; accessed November 2022) (57).

### *In vitro* filter mating assays.

In order to evaluate the transferability of the colistin resistance, filter mating assays were performed. Briefly, liquid cultures of *mcr*-carrying donor strains and the SAZ^r^ E. coli J53 recipient strain were mixed in a ratio of 1:2 and centrifuged at 3,500 × *g* for 5 min. The supernatant was discarded, and the remaining pellet was resuspended in 100 μL lysogeny broth (LB) and applied on a 0.2 μm pore-size filter on an LB agar plate. Following an incubation of 24 h at 37°C, bacteria were removed from pore-size filters by suspending in 4 mL LB broth. Transconjugants were selected by plating 100 μL aliquots on LB agar plates supplemented with 100 mg/L sodium azide and 2 mg/L colistin. To verify successful conjugation, the transconjugants were subjected to AST and analyzed for the presence of *mcr-1* and beta-lactamase (*bla*_TEM_) by PCR.

### PCR.

The presence of *mcr-1.26* and *bla*_TEM_ was determined by PCR as previously published (11, 58), while the proximity of both genes was confirmed as follows. First, genomic DNA of poultry isolates and corresponding transconjugants was analyzed for the presence of *bla*_TEM_ using the primers and PCR conditions as published resulting in an amplification product of 503 bp (see Fig. S3 in the supplemental material) (58). Subsequently, the intergenic region between *mcr-1.26* and *bla*_TEM_, including the genes, was amplified with the reverse primers (Mcr-1a REV 5′-GGGCATTTTGGAGCATGGTC-3′/TEM-R 5′-ACCAATGCTTAATCAGTGAG-3′; annealing temperature, 55°C; elongation time, 480 s), leading to a 7,317-bp product confirming the close proximity of the resistance genes (17, 59).

### Data availability.

The assembled genomic sequences of poultry isolates were deposited under the BioProject number PRJNA726012 in the NCBI database. The two plasmid sequences were deposited in GenBank (pEc141188, ID2678299, accession number OQ557085; pEC200574, ID2678314, accession number OQ557086). The Lipid A phosphoethanolamine transferase *mcr-1.26* is annotated as a misc feature due to the altered start codon.
