# Supplementary material for: Genomic Evidence of mcr-1.26 IncX4 Plasmid Transmission between Poultry and Humans
Source: Microbiol Spectr. 2023 Jun 26;11(4):e01015-23. doi: 10.1128/spectrum.01015-23 (PMC10434184; doi:10.1128/spectrum.01015-23)
Supplement: Supplemental file 1 — Fig. S1 to S4. Download spectrum.01015-23-s0001.docx, DOCX file, 1.6 MB [file spectrum.01015-23-s0001.docx]

**Supplemental Material**

**Genomic evidence of *mcr-1.26* IncX4 plasmid transmission between poultry and humans**

Ulrike Binsker,^a,#^ Kathrin Oelgeschläger,^a^ Bernd Neumann,^b^ Guido Werner,^c^ Annemarie Käsbohrer,^a,d^ and Jens A. Hammerl^a^

^a^Department Biological Safety, German Federal Institute for Risk Assessment, Berlin, Germany

^b^Institute for Hospital Hygiene, Medical Microbiology and Clinical Infectiology, Paracelsus Medical University, Nuremberg General Hospital, Germany

^c^Department of Infectious Diseases, Robert Koch Institute, Wernigerode, Germany

^d^Department for Farm Animals and Veterinary Public Health, Institute of Veterinary Public Health, University of Veterinary Medicine Vienna, Vienna, Austria

Running title: Similar *mcr-1.26* IncX4 plasmids in poultry and humans

#Address correspondence to Ulrike.Binsker@bfr.bund.de

Address: Unit Epidemiology, Zoonoses and Antimicrobial Resistance, Department Biological Safety, German Federal Institute for Risk Assessment, Diedersdorfer Weg 1, 12277 Berlin, Germany

Telephone: +49 30 18412 24340

**Figure S1**

**Figure S1: Phenotypic antimicrobial susceptibility profiles from *mcr-1.26* positive *E. coli* isolates from poultry in Germany.**

Microbiological resistance profiles of 16 *E. coli* were determined using the broth microdilution method according to CLSI guidelines following EUCAST epidemiological cut-off values. For quality assurance purposes, the *E. coli* isolate ATCC 25922 has been included as a reference in the AST measurements. AK: amikacin, AMP: ampicillin, FOT: cefotaxime, TAZ: ceftazidime, CHL: chloramphenicol, CIP: ciprofloxacin, COL: colistin, GEN: gentamicin, MERO: meropenem, NAL: nalidixic acid, SMX: sulfamethoxazole, TET: tetracycline, TGC: tigecycline, and TMP: trimethoprim.

**Figure S2**

**
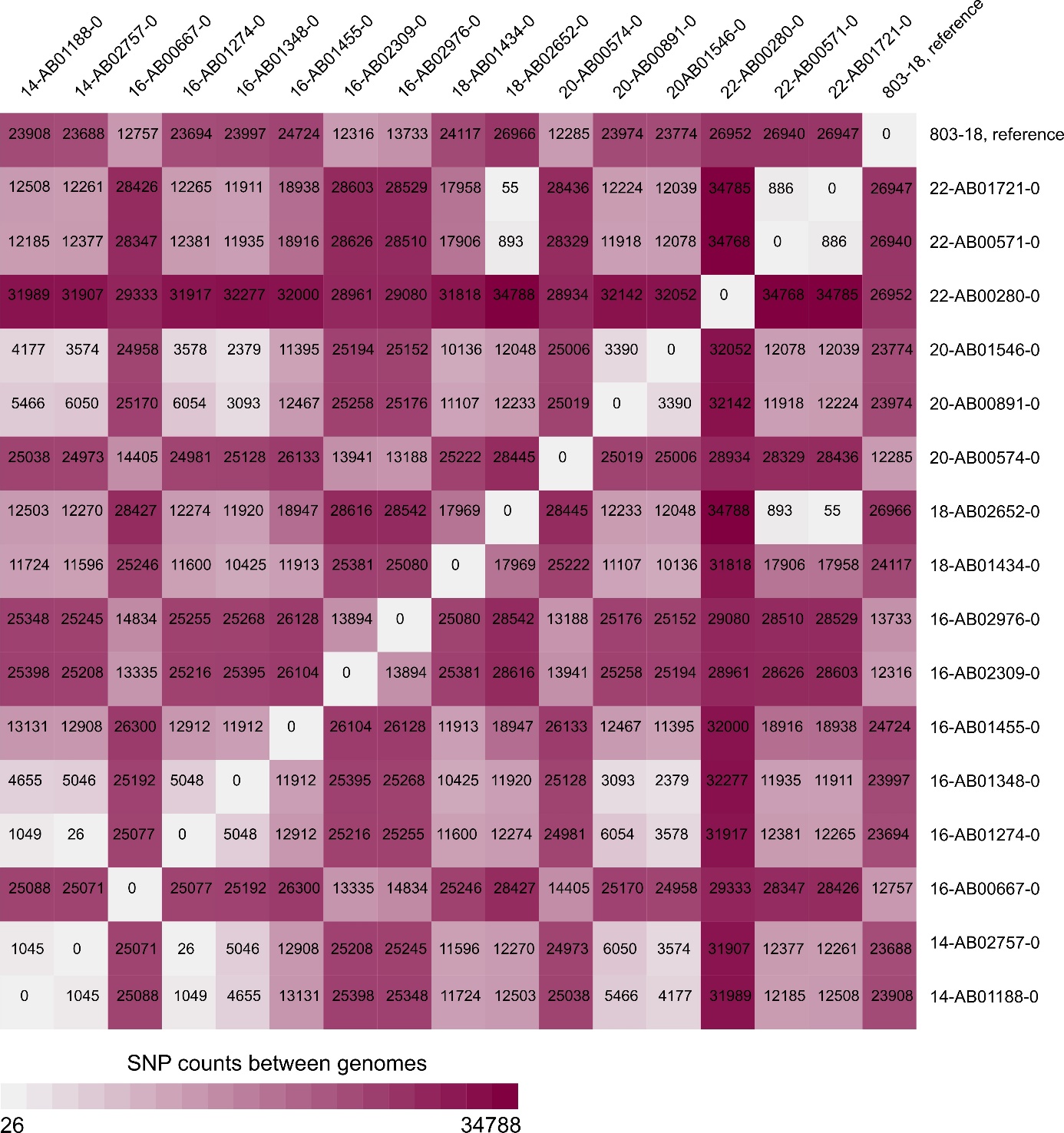
**

**Figure S2:** **Heat map of SNP differences of *mcr-1.26*-positive *E. coli* from poultry and human clinical samples in Germany.**

Pairwise distance matrix based on SNP was obtained with the web-based tool CSIPhylogeny 1.4 (default settings) of the Center for Genomic Epidemiology (CGE) (www.genomicepidemiology.org) using the human clinical isolate 803-18 as reference. SNP distances are colored from minimum (white) to maximum (dark red).

**Figure S3**


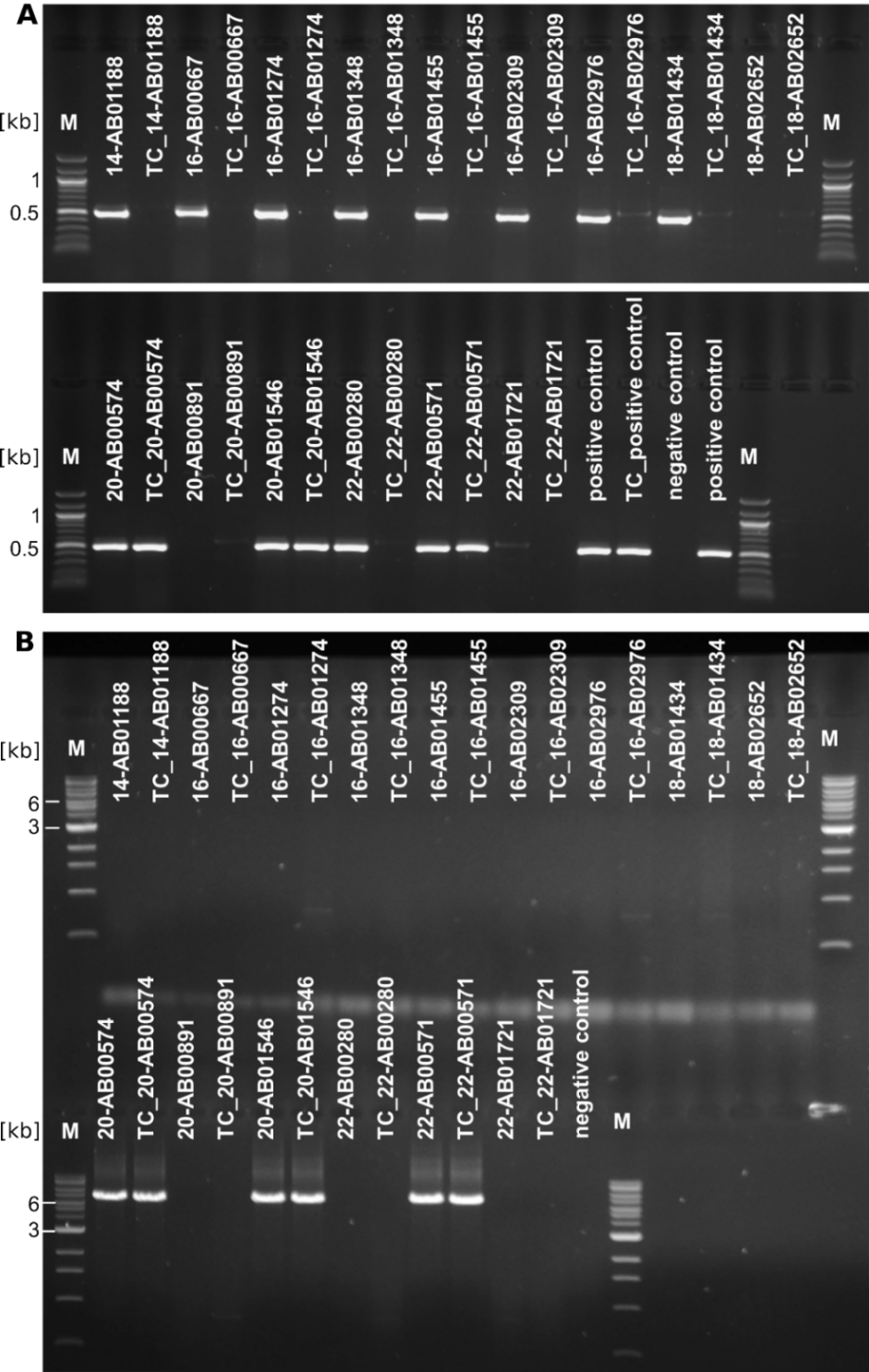


**Figure S3: Detection of the adjacent localization of *mcr-1.26* and *bla*_TEM_ in *E. coli* wildtype strains and corresponding transconjugants.**

PCR was performed as described in the method section. A) Amplification of *bla*_TEM_ in isolates and transconjugants (TC) leading to a 503 bp product, Marker: Quick-Load® 100 bp DNA Ladder. B) Amplification of the intergenic region between *mcr-1.26* and *bla*_TEM_ in isolates and transconjugants (TC) resulting in a 7,317 bp product in 20-AB00574, 20-AB01546 and 22-AB00571. Marker: Quick-Load® 1 kb Extend DNA Ladder (NEB). Negative control: genomic DNA of SAZ^R^ *E. coli* J53.

**Figure S4**

**
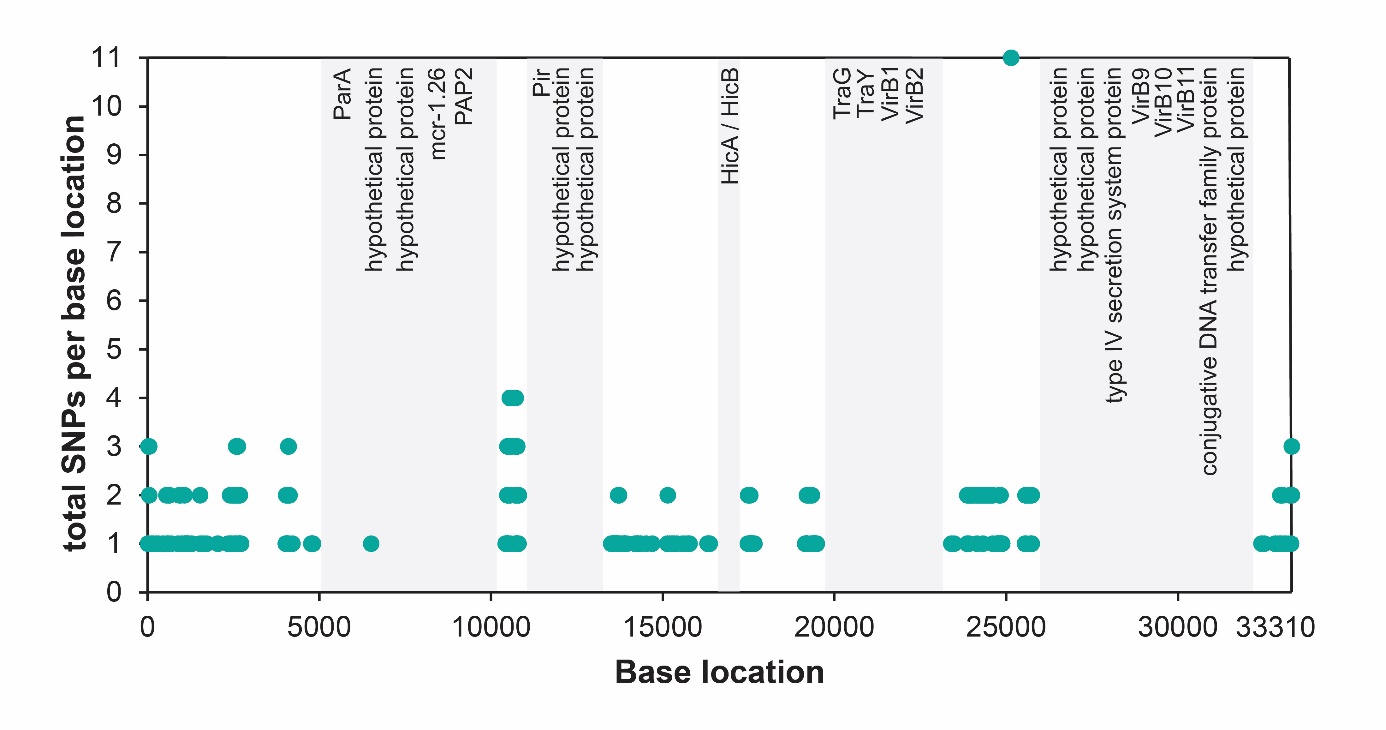
**

**Figure S4: SNPs in *mcr-1.26* IncX4 plasmids of all poultry isolates.**

Total number of SNPs in protein coding sequences of all isolates using pEC141188 as reference. Names of highly conserved coding sequences without SNPs are shown and marked with a grey background. SNPs in intergenic regions are not shown.
